# Supplementary figures and images for: Sequencing and analysis of 131 SARS-CoV-2 isolates in previously sampled and unsampled regions of Jordan from 2020 to 2023
Source: PLoS One. 2025 Oct 27;20(10):e0335070. doi: 10.1371/journal.pone.0335070 (PMC12558501; doi:10.1371/journal.pone.0335070)

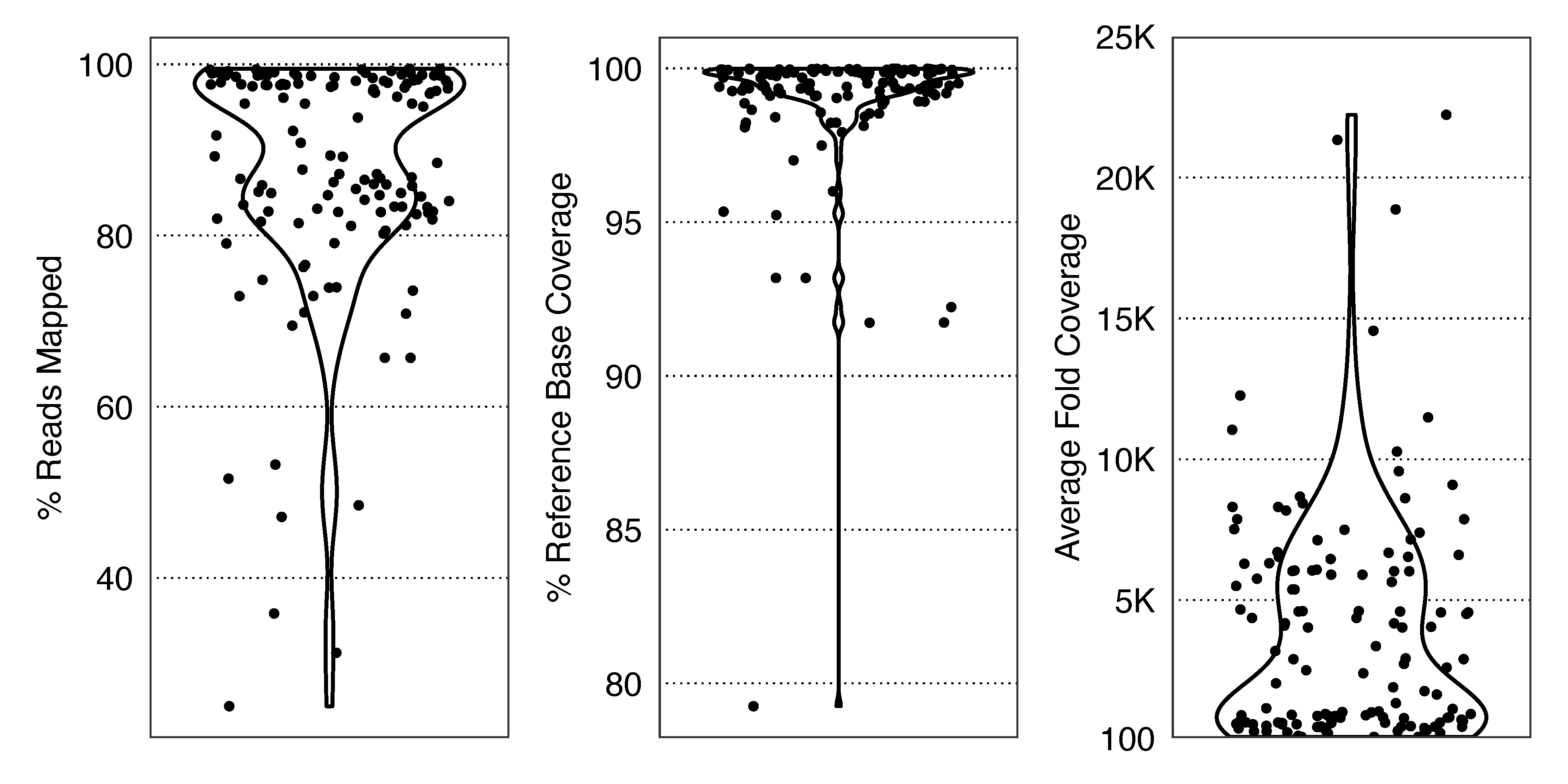

Supplement: S1 Fig — Statistics describing alignment performance of cleaned reads against the reference SARS-CoV-2 Wuhan-Hu-1 complete genome. Induvial dots describe the performance of samples for percentage of total cleaned reads mapped to the reference (left), the percent of reference bases covered by one or more sample reads (middle), and the average fold coverage of reads mapped against the reference (right). (TIFF) [file pone.0335070.s001.tiff]
