# Supplementary material for: Sequencing and analysis of 131 SARS-CoV-2 isolates in previously sampled and unsampled regions of Jordan from 2020 to 2023
Source: PLoS One. 2025 Oct 27;20(10):e0335070. doi: 10.1371/journal.pone.0335070 (PMC12558501; doi:10.1371/journal.pone.0335070)
Supplement: Attachment — Submitted filename: Response to Reviewers.docx [file pone.0335070.s004.docx]

|  | Los Alamos National Laboratory  PO Box 1663, G723  Los Alamos, NM 87545  707-812-3531 | |
| --- | --- | --- |
| B-GEN: Genomics and Bioanalytics  Bioscience Division | **Date:** | 4 November 2024 |
|  | | |

Nihad A.M Al-Rashedi
Academic Editor
PLOS ONE

Dear Dr. Al-Rashedi and Reviewers:

I write to resubmit our manuscript entitled “Sequencing and analysis of 131 SARS-CoV-2 samples in Jordan highlight cooperative partnerships in developing NGS capabilities” to *PLOS ONE* for publication as an original research article. Thank you for providing us a chance to revise our manuscript. We have paid close attention to the comments by each reviewer and have responded to each one. We have also formatted the manuscript to PLOS ONE’s style. Please note that in addition to copying our responses in the box provided in the online submission portal, we have pasted our responses (in bold) below. We also tracked each change in the anonymized manuscript using track changes and uploaded a clean version of the non-anonymized manuscript to the submission portal.

We would like to point out major changes that were made during the revision process. We significantly reduced the emphasis on collaboration in analyzing these sequences from Jordan and better describe the overall patterns of SARS-CoV-2 sequences over time. Because of this, we changed the title slightly to better reflect the results of the paper. Additionally, we believe that this should be published as a regular article in PLOS ONE even though Reviewer 2 suggested it be a short communication given the small sample size. The addition of 131 genomes of SARS-CoV-2 sequences in Jordan is quite an achievement and the added analyses will help their country, and the rest of the Middle East, learn from this pandemic and apply these lessons to future outbreaks.

Regarding the comments to the journal-specific requirements, we would like to declare an amended Role of Funder statement as follows: The funders had no role in study design, data collection and analysis, decision to publish, or preparation of the manuscript. We have also amended our acknowledgement section as requested to remove all funding-related information. It now reads: “We would like to thank the Defense Threat Reduction Agency – Cooperative Threat Reduction Program for facilitating collaboration and communication between partner organizations. We also thank Cheryl Gleasner and the rest of the LANL Sequencing team for the NGS guidance provided to Royal Medical Services throughout sequencing efforts”.

This is an original manuscript. It has not been published before and is not being considered for publication in another journal. All authors have approved the final manuscript for submission, and we declare no competing interests. Thank you for considering this manuscript for publication in *PLOS ONE*. We look forward to hearing from you again.

Sincerely,

Samuel I. Koehler

Research Technologist

Biosecurity and Public Health

Los Alamos National Laboratory

Email: sikoehler@lanl.gov

Phone: +1 (707) 812-3531

Response to Reviewers, Journal Requirements

1. When submitting your revision, we need you to address these additional requirements.

Please ensure that your manuscript meets PLOS ONE's style requirements, including those for file naming.

**Thank you for bringing this to our attention. Headers have been applied to appropriate section titles throughout the manuscript and file names have been revised to match the required formatting.**

2. We note that the grant information you provided in the ‘Funding Information’ and ‘Financial Disclosure’ sections do not match. When you resubmit, please ensure that you provide the correct grant numbers for the awards you received for your study in the ‘Funding Information’ section.

**Thank you for identifying this discrepancy. We have updated the Funding Information section to reflect both DTRA and DOE-LANL awards.**

3. Thank you for stating the following financial disclosure:

[This material is based upon work supported by the Cooperative Threat Reduction (CTR) Biological Threat Reduction Program (BTRP) under Award/Contract No. HDTRA1343361to Triad National Security, LLC, an operator of the Los Alamos National Laboratory under contract No. 89233218CNA000001 with the US Department of Energy.].

Please state what role the funders took in the study.  If the funders had no role, please state: "The funders had no role in study design, data collection and analysis, decision to publish, or preparation of the manuscript."

If this statement is not correct you must amend it as needed. Please include this amended Role of Funder statement in your cover letter; we will change the online submission form on your behalf.

**Thank you for clarifying this section. We would like to declare an amended Role of Funder statement as follows: “The funders had no role in study design, data collection and analysis, decision to publish, or preparation of the manuscript”.**

4. Thank you for stating the following in the Acknowledgments Section of your manuscript:

[This material is based upon work supported by the Cooperative Threat Reduction (CTR) Biological Threat Reduction Program (BTRP) under Award/Contract No. HDTRA1343361 to Triad National Security, LLC, an operator of the Los Alamos National Laboratory under contract No. 89233218CNA000001 with the US Department of Energy. ]

We note that you have provided funding information that is not currently declared in your Funding Statement. However, funding information should not appear in the Acknowledgments section or other areas of your manuscript. We will only publish funding information present in the Funding Statement section of the online submission form.

Please remove any funding-related text from the manuscript and let us know how you would like to update your Funding Statement. Currently, your Funding Statement reads as follows:

[This material is based upon work supported by the Cooperative Threat Reduction (CTR) Biological Threat Reduction Program (BTRP) under Award/Contract No. HDTRA1343361to Triad National Security, LLC, an operator of the Los Alamos National Laboratory under contract No. 89233218CNA000001 with the US Department of Energy.].

Please include your amended statements within your cover letter; we will change the online submission form on your behalf.

**Thank you for clarifying the contents of the Acknowledgement section for us. We amended our Acknowledgement section as requested to remove all funding-related information. It now reads: “We would like to thank the Defense Threat Reduction Agency – Cooperative Threat Reduction Program for facilitating collaboration and communication between partner organizations. We also thank Cheryl Gleasner and the rest of the LANL Sequencing team for the NGS guidance provided to Royal Medical Services throughout sequencing efforts”.**

5. Please provide a complete Data Availability Statement in the submission form, ensuring you include all necessary access information or a reason for why you are unable to make your data freely accessible. If your research concerns only data provided within your submission, please write "All data are in the manuscript and/or supporting information files" as your Data Availability Statement.

**Thank you for bringing this to our attention. The submission form has an updated Data Availability Statement clarifying how to access samples, as well as stating why low-quality genomes were excluded from upload and analysis while their associated metadata is readily available.**

6. Please include your full ethics statement in the ‘Methods’ section of your manuscript file. In your statement, please include the full name of the IRB or ethics committee who approved or waived your study, as well as whether or not you obtained informed written or verbal consent. If consent was waived for your study, please include this information in your statement as well.

**Thank you for identifying this issue with our Ethics Statement. We have now included a full Ethics Statement in the manuscript Methods sub-section ‘Ethical clearance’ as well as updated the online submission sections on Data Availability to reflect these changes.**

7. We are unable to open your Supporting Information file [Table S1.tsv]. Please kindly revise as necessary and re-upload.

**Thank you for identifying this issue. Supporting Information file [Table S1.tsv] has been re-uploaded.**

____________________________________________________________________________________

Response to Reviewers, Reviewer #1

Major comments:

1. The sample size is relatively small (131 genomes) compared to many published SARS-CoV-2 genomic epidemiology studies. The authors should more clearly acknowledge this limitation and discuss how it may impact the conclusions that can be drawn

**Thank you for bringing this to our attention. We agree that our sample size is relatively small and have revised many sections (Abstract, Introduction, Discussion) to properly contextualize our framing and interpretation of the results.**

2. The phylogenetic analysis and discussion of lineage dynamics over time could be expanded. More detail on how the observed patterns compare to regional and global trends would provide valuable context.

**Thank you for bringing this to our attention. We have expanded a paragraph in the Discission expanded to more thoroughly compare our findings to existing global and Jordan covid-19 pandemic phylogenetic reporting.**

3. The statistical analysis of symptoms and vaccination status (lines 265-271) is limited by small sample sizes in some groups. The authors should be more cautious in drawing conclusions from these analyses and clearly state the limitations.

**Thank you for pointing this out. We decided to remove this analysis from the manuscript to avoid over stating the importance of these results based on small sample sizes. We still describe the vaccination patterns qualitatively. We also left in the logistic regression of SARI symptoms and vaccine type. This is a**

4. The discussion of the utility of the EDGE-COVID-19 platform feels somewhat disconnected from the rest of the manuscript. This section could be condensed and better integrated with the main findings.

**Thank you for identifying this disconnection. We have revised the manuscript to better contextualize EDGE in relation to the development of new NGS capabilities and how the platform can leverage this objective. Many sections have been re-written to incorporate this.**

Minor comments:

5. Lines 79-91: The background on Jordan's healthcare system and involvement in COVID-19 response is useful but feels somewhat out of place in the introduction. Consider moving some of this information to the methods or discussion.

**Thank you for this helpful comment. This section has been moved from the introduction to the end of the discussion.**

6. More details on the quality control criteria used to exclude samples from phylogenetic analysis would be helpful.

**We agree that the quality control criteria was lacking. The section “Phylogenetics” has been expanded to better describe this.**

7. Figure 1: The color scheme in panel C is difficult to interpret. Consider using a more color-blind friendly palette.

**Thank you for pointing this out. Figure 1.C has been revised to be more color-blind friendly.**

Response to Reviewers, Reviewer #2:

Major comments:

1. The authors are trying to showcase the outcome is the collaborative research between two countries which enable NGS-based sequencing and bioinformatic analyses. Publication in research journal should merit research outcome i.e. the results and discussion but not the collaborative efforts. This is not a platform for this purpose. Unless the authors willingly remove credits on the collaboration and emphasize only on the results and discussion, this manuscript could then be considered for publication in a scientific journal.

**We thank the reviewer for bringing this to our attention. Our primary research focus here was to report on the sequencing and analysis of 131 covid sequences produced in Jordan. This quantity of samples represents a significant proportion for Jordan SARS-CoV-2 genomes and includes many samples collected from novel regions at dates not previously reported. To this end, we strongly believe that this work constitutes scientific outcomes worthy of publication with PLOS ONE. We agree with the reviewer that the focus of publication is not to showcase collaboration, and to this end we have significantly reduced emphasis on collaboration. Limited discussion of collaboration remains in the text, as we believe that demonstrating collaboration as a means towards developing NGS capability in a country with minimal sequencing productivity represents an important milestone worth highlighting.**

2. The sample size is too small to represent a country. This manuscript should go for a short communication or brief report.

**Thank you for identifying this limitation. A similar comment was made up by Reviewer #1 and has been addressed in detail, see responses to Reviewer #1 comments 1-3. We believe that while small, our samples and reporting represent an important contribution to the SARS-CoV-2 reporting for a country that is developing its NGS capabilities and has relatively few published genomic resources available in databases. This work represents a major contribution to the total available SARS-CoV-2 genome sequences collected from within Jordan, notably at many locations and dates that have not yet been reported.**

Minor comments:

1. Should provide the GISAID ID i.e. uploaded and accepted sequences in the manuscript, instead of the submission form to GISAID (suppl file).

**The section “Bioinformatics” was revised to describe how uploaded and accepted sequences may be accessed on GISAID.**

2. The data analyses should be specific. It mentioned that the sequence reads were mapped to the reference genome. But the subsequent description revealed that the genomic sequences used are assembled (de novo?).

**Thank you for this comment. The data analysis methods in the “Bioinformatics” and “Phylogenetics” sections have been revised to specify our methods in greater detail.**

3. Do you mean some of the assembled genomes only cover 65% of the reference genome, or reads mapping rate is 65%? I prefer only assembled genome sequences at complete and near complete to be used for subsequent analyses to avoid loss of data and inaccurate assignment of lineage and interpretation of prevalence of strains.

**Thank you for identifying this issue for clarification. Our statement that “a handful of samples reporting read mapping rates below 60%...” refers to the percentage of total raw reads, with the excluded 40% of reads being removed due to human host removal. Minimum linear genome coverage achieved in this analysis was 79%, and only samples with 95% or greater linear coverage were included in the phylogenetic analysis. This section has been revised to more clearly describe this. The “Bioinformatics” section now also clarifies that only samples with >95% linear coverage was used.**
