# Supplementary material for: Sequencing and analysis of 131 SARS-CoV-2 isolates in previously sampled and unsampled regions of Jordan from 2020 to 2023
Source: PLoS One. 2025 Oct 27;20(10):e0335070. doi: 10.1371/journal.pone.0335070 (PMC12558501; doi:10.1371/journal.pone.0335070)
Supplement: Attachment — Submitted filename: Response_to_Reviewers.docx [file pone.0335070.s005.docx]

_____________________________________________________________________________________

Response to Reviewers, Reviewer #1 Major Comments

1. Contribution to Genomic Surveillance:

- Strength: The study provides valuable genomic data from Jordan, especially from under-sampled regions, contributing to the global understanding of SARS-CoV-2 evolution.
- Suggestion: The manuscript could further highlight how these new sequences fill specific gaps in the global databases and their potential impact on public health strategies in Jordan.

**Thank you for suggesting that we further highlight the gaps that our sequences may fill. Efforts to combat future pandemics are informed by studying how we responded to recent ones like Covid-19. To effectively study our response to SARS-CoV-2, we need comprehensive databases and meta data (such as government response information) to draw conclusions from. A primary benefit of our sequencing is the expansion of timepoints and previously unreported strain identifications in Jordanian cities with few existing samples. While our samples are not enough to be able to broadly inform public health strategies, it will lend additional statistical power to any future global or regional analyses that include Jordan. One simple health strategy revealed by our analysis is the benefit of even small (131 samples) sequencing efforts that can reveal previously unreported strain information. This information has been incorporated into the discussion section at lines 340 - 348.**

2. Depth of Phylogenetic Analysis:

- Strength: The authors have expanded their phylogenetic analysis to compare findings with global and regional trends.
- Suggestion: A more detailed interpretation of the phylogenetic tree could be provided. Discussing possible transmission pathways, introductions of variants, and the implications for local transmission dynamics would strengthen the analysis.

**Thank you for requesting a more detailed interpretation of the phylogenetic analysis. While we lack much sequencing data from the important early years of the pandemic (2020, 2021), we can further interpret strain presence and distribution in 2022 and 2023 and how that may be explained by events within the country. This interpretation has been added throughout the discussion section at lines 426 – 430, 434 – 437, 440 – 450, 488 – 490.**

3. Integration of the EDGE Platform Discussion:

- Strength: The revised manuscript better integrates the discussion of the EDGE COVID-19 platform.
- Suggestion: Providing specific examples of how the platform facilitated the research and any challenges faced would offer practical insights into its utility in resource-limited settings.

**Thank you for suggesting that we provide examples of how EDGE COVID-19 facilitates research in limited settings. Bioinformatics analysis is often a bottleneck in genomic science as it requires intensive computation, which we did not readily have access to through our own servers. EDGE removed this bottleneck by providing free computational resources. Another strength of using EDGE COVID-19 was that it provided a suite of software specifically tailored for SARS-CoV-2. This specificity made it easier to understand and use the website for our analysis. The above information has been incorporated into our discussion of EDGE COVID-19 at lines 462 – 472.**

4. Analysis of Symptoms and Vaccination Status:

- Strength: The authors appropriately removed statistical analyses limited by small sample sizes.
- Suggestion: The qualitative descriptions could be enhanced by comparing them with national vaccination campaigns and existing literature to provide context and possible explanations for observed patterns.

**Thank you for identifying that qualitative descriptions could be enhanced by caparisons to national trends and existing literature. Information on Jordan’s national vaccination campaign, initiated by the Ministry of Health in mid-December 2020, has been incorporated into the discussion at lines 395 – 398, 413 - 418. Additionally, discussion of qualitative patient patterns has been supplemented with additional references to existing literature.**

5. Clarity and Organization:

- A thorough proofreading is necessary to improve clarity and readability.

**Thank you for this suggestion, we have reviewed the manuscript and have improved clarity and readability throughout.**
